# Supplementary figures and images for: n-3 and n-6 Polyunsaturated Fatty Acids Modulate Macrophage–Myocyte Inflammatory Crosstalk and Improve Myocyte Insulin Sensitivity
Source: Nutrients. 2024 Jun 29;16(13):2086. doi: 10.3390/nu16132086 (PMC11243049; doi:10.3390/nu16132086)

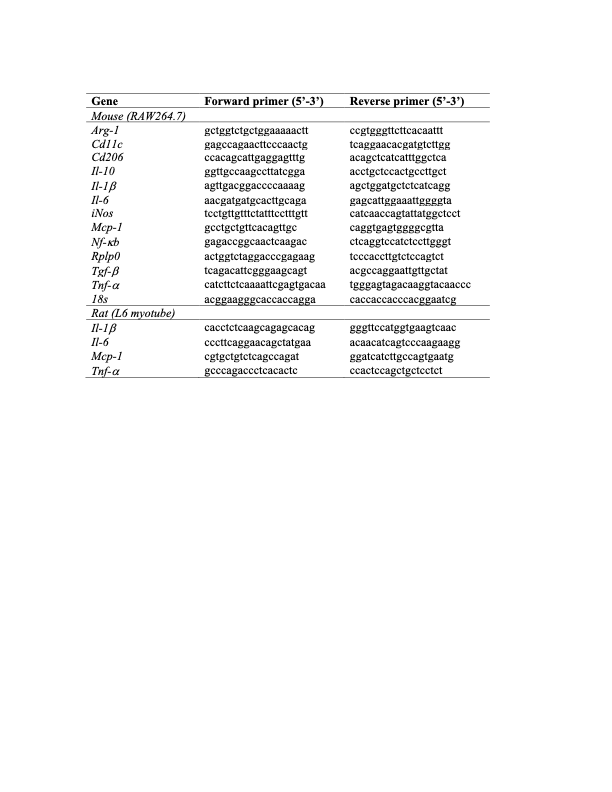

Supplement: Supplementary file 1 [file nutrients-16-02086-s001.zip › nutrients-3073267-supplementary/S1 Table.tiff]

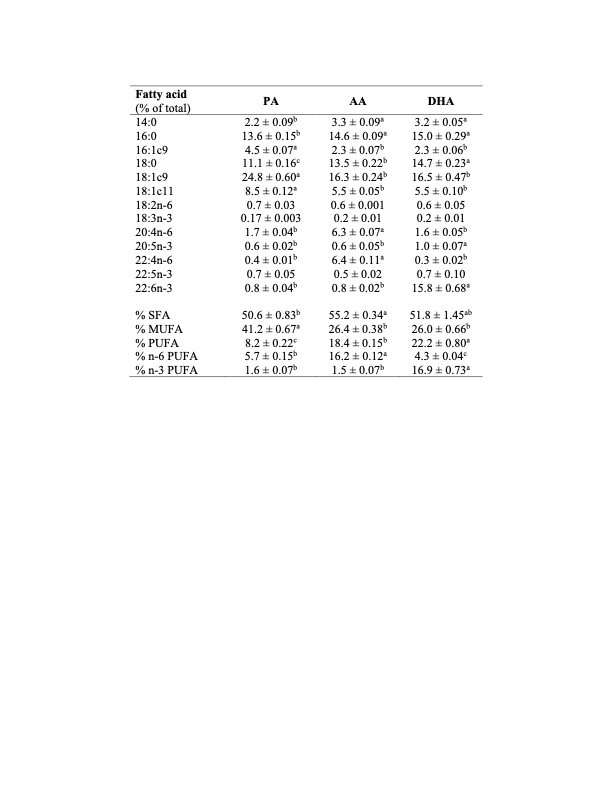

Supplement: Supplementary file 1 [file nutrients-16-02086-s001.zip › nutrients-3073267-supplementary/S2 Table.tiff]
